# Protocol for a randomized trial of a scalable, interactive tool to support surrogate decision-makers of critically ill patients

**DOI:** 10.1016/j.cct.2026.108262

**Published:** 2026-02-21

**Authors:** Aleksandra E. Olszewski, Rachel A. Butler, Deepshikha C. Ashana, Shannon Carson, Christopher E. Cox, Catherine L. Hough, David Y. Hwang, David Maloney, Florian Mayr, Vidya Menon, Jay S. Steingrub, Donald R. Sullivan, Grace Vincent, Blair Wendlandt, Douglas White

**Affiliations:** a Department of Critical Care Medicine, University of Pittsburgh, 3550 Terrace St, Pittsburgh, PA 15213, USA; b Department of Medicine, Duke University, Davison Building, 40 Duke Medicine Cir Room 401, Durham, NC 27710, USA; c Division of Pulmonary Diseases and Critical Care Medicine, Department of Medicine, University of North Carolina at Chapel Hill, 4th Floor, Bioinformatics Building, 130 Mason Farm Rd, Chapel Hill, NC 27514, USA; d Division of Pulmonary and Critical Care Medicine, Oregon Health & Science University, Physicians Pavilion, 3270 SW Pavilion Loop, Portland, OR 97239, USA; e Division of Neurocritical Care, Department of Neurology, University of North Carolina School of Medicine, 170 Manning Dr, Chapel Hill, NC 27599, USA; f Department of Medicine, Lincoln Medical Center - New York City Health and Hospitals, 234 E 149th St, Bronx, NY 10451, USA; g Department of Critical Care Medicine, University of Massachusetts Chan Medical School, Baystate, 759 Chestnut Street, Springfield, MA 01199, USA; h Division of Pulmonary Disease and Critical Care, Department of Medicine, University of North Carolina School of Medicine, 4th Floor, Bioinformatics Building, 130 Mason Farm Rd, Chapel Hill, NC 27514, USA

**Keywords:** Intensive care, Palliative care, Surrogate decision-making, Quality of communication, Patient-centered care, Clinical trial

## Abstract

**Background:**

Patients, particularly those at the end of their lives, frequently receive goal-discordant care, and their surrogate decision-makers suffer long-term psychological injury. Contributors to these issues may include infrequent communication between clinicians and surrogates, failure to discuss prognosis, values, and treatment options that include comfort-focused care, and surrogates facing high-stakes decision-making while underprepared and overwhelmed psychologically and emotionally.

**Design:**

This is a multicenter, patient-randomized efficacy trial of a multi-component intervention, versus usual care, for 370 incapacitated, critically ill adults at high risk of death or severe disability, and their surrogate decision-makers, from 7 hospitals across the United States.

**Intervention:**

The intervention combines surrogate utilization of a digital Family Support Tool (FST) in real-time during their loved one’s hospitalization with proactively scheduled family meetings, for which both surrogates and clinicians receive additional preparation, at set intervals during the ICU hospitalization. Those in the control arm will receive usual ICU care.

**Outcomes:**

Our primary outcome is patient-centeredness of care, measured using the modified Patient Perceived Patient-Centeredness of Care (PPPC) scale. Secondary outcomes include surrogates’ psychological symptom burden, communication and decision quality, and patients’ health resource utilization and clinical outcomes.

**Conclusion:**

This trial will provide robust evidence about the impact of combining the FST with increased and intentional communication, on patient, family, and health system outcomes for those hospitalized in the ICU.

## 1. Background

Up to a quarter of the time, patients receiving care toward the end of their life receive treatment that is inconsistent with their values and preferences, typically comprising more invasive and burdensome therapies than they would choose for themselves [1–3]. At the same time, medical care at the end of a patient’s life is a key contributor to health care costs, accounting for 25% of Medicare costs [4]. Regardless of whether patients are facing the end of their life or other challenges related to critical illness, family members faced with serving as surrogate decision-makers for their loved ones suffer psychological distress (including lasting psychological harms such as depression, anxiety, and post-traumatic stress disorder), guilt, and doubt about their decisions [5–9]. Multiple factors contribute to these growing problems, including (1) infrequent communication between clinicians and surrogates, (2) frequent failure to discuss prognosis, values, and treatment options that include comfort-focused care, and (3) surrogates facing high-stakes decision-making while underprepared and overwhelmed psychologically and emotionally.

We sought to design a scalable, in-the-moment, feasible intervention that combined an interactive digital tool with systems-level care delivery changes in order to attend to these three mechanisms contributing to goal-discordant care and surrogate psychological harm in critical illness. Our goals were to design an intervention that is easy to use, scalable to diverse health systems, and applicable to a broad range of diseases that cause critical illness [10–14]. Existing evidence shows that family support interventions delivered by trained interventionists can improve outcomes [15,16]. However, such interventions require adding people to the ICU team, making these interventions expensive and difficult to scale due to the amount of training required [9,17,18]. We therefore developed a multi-component intervention that involves increased targeted communication between existing clinicians and families via proactive family meetings scheduled at set intervals, unlimited access to an interactive digital tool (the Family Support Tool, or FST) for family emotional support and decisional preparation, and feedback to clinicians in advance of family meetings regarding family questions, expectations about prognosis and treatment leaning. We previously conducted pilot testing of the web-based FST, followed by a two-arm, patient-level randomized pilot trial comparing the FST to enhanced usual care, establishing feasibility of recruitment and retention (with 96% of enrolled participants using the tool per protocol), as well as receiving excellent ratings by surrogates of acceptability, usability, and effectiveness. We will conduct a randomized trial to determine whether the multi-component intervention can improve (1) patient clinical outcomes, (2) surrogate long-term psychosocial outcomes, (3) decision and communication quality, and (4) end-of-life healthcare utilization. We hypothesize that, compared to standard ICU care, the intervention will result in increased patient-centeredness of care, decreased surrogate psychological symptom burden and decreased duration and intensity of treatment at the end of life.

## 2. Methods

### 2.1. Trial design

This is a multicenter, patient-randomized efficacy trial, funded by the National Institute on Aging (grant number R01AG06673), approved by the University of Pittsburgh Institutional Review Board (IRB; STUDY20110367), and registered on ClinicalTrials.gov (trial number NCT05019261). This trial follows the CONSORT guidelines for reporting non-pharmacological treatment interventions [19].

### 2.2. Study setting

Participants will be enrolled from 20 ICUs at 7 sites across the United States, ensuring a diverse patient population and including both community and academic hospitals. All included sites have access to palliative care, and no included sites have protocolized approaches to family communication.

### 2.3. Eligibility criteria

We will recruit 370 incapacitated, critically ill adults at high risk of death or severe disability, along with their surrogate decision-makers and their attending physician or the attending’s designee. Participants will be recruited within the first 5 days of ICU admission. Patient eligibility criteria will include age ≥ 18 years, lack of decision-making capacity as determined by clinical examination by the attending physician or designee, and clinical indication of at least 40% risk of death, or ≥ 40% chance of new, severe long-term functional impairment (needs assistance with ≥2 activities of daily living) as judged by the patient’s attending physician or designee. We will enroll one primary surrogate and up to 3 additional surrogates per patient (an average of 1.5 surrogates per patient), with the primary surrogate being decided by the patient’s advance directive or by following the hierarchy of surrogates codified in state law. Surrogates are eligible for the study both in-person and remotely. Measures to support remote participation by surrogates include:

1. Access to FST using a personal electronic device of their choosing, with study staff checking out a device for those who do not have a device, to be used at the hospital.
2. If surrogates are unable to make it to the hospital, study staff will work to identify access to a device via family members or publicly available devices or computers (e.g. at a library).
3. Family meetings may take place in-person, via conference call, or video meeting.
4. If surrogates express difficulty completing sections of the FST, study staff will work with them either in person or remotely, depending on their ability to visit the hospital and their access to technology.

Patient exclusion criteria include lack of surrogate decision maker, family not available for study, imminent death (within 24 h), goals of care already “comfort measures only,” decision for life support withdrawal already made, current participation in competing research study, incarceration or involuntary hold, death prior to enrollment, discharge prior to enrollment, regaining of capacity prior to enrollment, physician and/or designee declining participation, more than 5 days of ICU hospitalization. Surrogate exclusion criteria include being <18 years old, not having access to a device with internet capabilities, being unable to read or understand English, and having cognitive or physical limitations impacting ability to complete study questionnaires.

### 2.4. Participant screening, recruitment and consent

A member of the research team will screen each ICU daily to identify patients meeting eligibility criteria, as we have established in prior work [20–22]. Surrogates will give proxy consent for incapacitated patients. For patients who regain capacity during the hospitalization, we will perform re-consenting.

### 2.5. Intervention

**Fig. 1.**
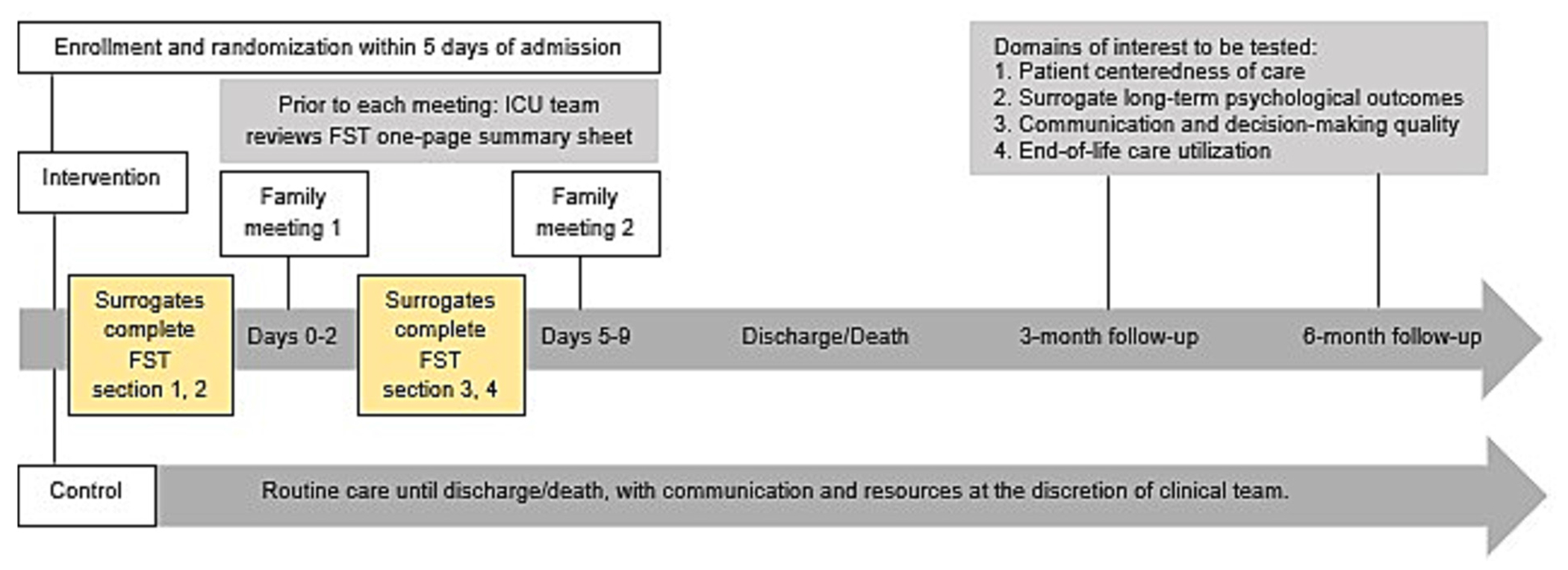
Steps involved in the trial, starting with study enrollment.

The study intervention combines 1) proactively scheduled family meetings (to increase and standardize mechanisms for communication), 2) the interactive, web-based FST for surrogates (to provide surrogate emotional support and increase decisional preparedness and understanding, as well as improve quality of communication), and 3) in-the-moment feedback and information shared with clinical teams in the form of a printed report generated by the surrogates’ responses to FST prompts and exercises that summarizes the family’s main questions and concerns, information about the patient’s values and preferences, surrogates’ prognostic expectations, and a visual display of their unmet needs (Fig. 1, Table 1).

The FST uses videos and animation, as well as narration by actor family members of ICU patients. The Cognitive Emotional Decision-Making (CEDM) Framework, the Ottawa Decision Support Framework, and input from family- and clinician stakeholders all informed the intervention’s process changes and FST (Table 1) [14,17,18]. The CEDM emphasizes the emotional and psychological influencers on decision-making, in addition to cognitive and logical ones. Families who have served as surrogates for patients hospitalized in the ICU, including patient families who faced a mix of patient outcomes (survival, different degrees of recovers, and loss of life), recorded video messages of support, communication tips, stories about their experiences and coping strategies; clinicians recorded videos of encouragement; and self-care tips were all included in the FST in order to attend to the components of this framework. The Ottawa Decision Support Framework is an evidence-based tool that supports individualized patient and family decision-making [14,17,18]. The FST includes videos explaining ICU care, routines, machines, and personnel and videos preparing surrogates for family meetings, and describing three broad potential pathways of care (comfort-focused treatment, life-extending treatment, or trial of life-extending treatment), in order to attend to key components of this framework. In addition, the FST incorporates the Ottawa Decision Support Framework with interactive exercises, prompts, and direct questions about the patient’s preferences, values and treatment leanings, which will be then shared with the clinical team.

Upon enrollment, surrogates will be oriented to the FST with a handout and with the study team. Surrogates in the intervention arm will receive unlimited access to the FST throughout hospitalization. Study staff will liaise with the enrolled surrogates and ICU team to schedule the first family meeting, within 2 days of enrollment (Table 1, Fig. 1). Family meetings may take place in person, via conference call, or via video meeting and must include the enrolled surrogates and ICU attending or their designee. Additional meeting participants will be at the discretion of the enrolled surrogates and attending. The tool was designed to be used sequentially in sections of approximately 25 min each, yet with time periods for reflection and processing interspersed. In addition, family members can review any useful sections at any time. Surrogates will be prompted to use the tool upon enrollment (section 1: about the ICU), and before the first clinician-family meeting (section 2: getting ready to talk to the healthcare team, section 3: your loved one’s values and treatment options). The study team will monitor completion and prompt family members as needed. After completing the FST, the study team will provide surrogates and ICU team members with a brief summary report containing information provided by the surrogates, such as their main questions, their preferred decision-making role, their loved one’s health-related values, their prognostic expectations and their understanding of their loved one’s treatment preference (i.e., toward life-extending care, a trial of life-extending care, or comfort-focused care). The study team will briefly review the summary with clinicians, which prompts them to address key elements of shared decision-making.

If the patient remains in the ICU on study day 5, study staff will liaise with surrogates and the ICU team to schedule the second family meeting, to take place on days 5–9 of enrollment. At least 24 h before the second scheduled family meeting, the study staff will assign the surrogates FST section 4: getting ready for your next meeting with the healthcare team. Study staff will similarly monitor surrogates’ progress and provide support for completion. On study day 5, the surrogates and ICU staff will be administered the day 5 survey (which takes 10–15 min), via REDCap, or administered by staff, assessing their understanding of their loved one’s prognosis, their opinions on the communication, the FST, and on their own decision-making. The clinician participating in the patient’s final family meeting (whether it is meeting 1 or meeting 2) will complete a brief survey (5–10 min) sharing their perspective on the patient’s clinical case and prognosis, as well as their opinions on the FST. If the patient remains in the ICU after the second family meeting, a weekly family meeting will be scheduled within 5–9 days of the previous meeting.

### 2.6. Implementation support

Prior to participant enrollment, the study team will work with ICU leadership to develop a feasible and acceptable process for coordinating family meetings with the enrolling units. Using methods informed by the pilot study, research staff will orient surrogates to the FST and be available for questions. The FST has a very simple interface and is accessible anytime and anywhere for surrogates via smartphone, tablet, or computer. If needed, surrogates had access to a tablet computer provided by research staff.

### 2.7. Intervention fidelity monitoring and maintenance

**Fig. 2.**
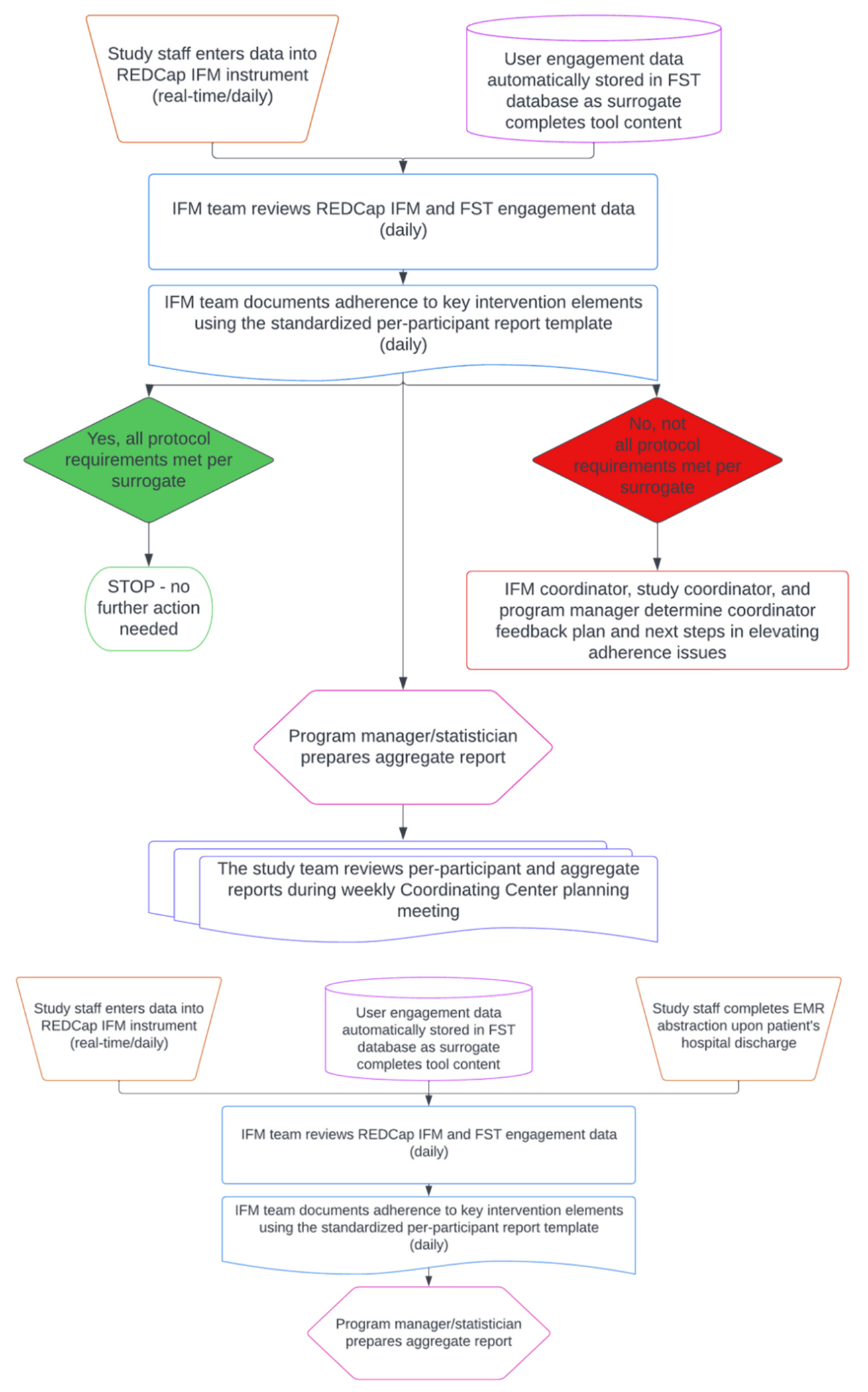
Intervention fidelity monitoring strategies. Daily or weekly strategies shown at the top of the figure, with monthly strategies shown below. IFM = intervention fidelity monitoring. FST = Family Support Tool.

We will adhere to best practice recommendations from the NIH Behavior Change Consortium [23], to establish, monitor, and maintain intervention fidelity (Fig. 2). To establish intervention fidelity, research staff will receive training (virtually via video conference) on the detailed manual of procedures. Trainings will consist of a series of didactic sessions that included presentations, discussions, and role play. We developed a rubric that will be used in these interactive sessions, during which participant-facing coordinators will receive feedback, and will be certified only once proficiency is demonstrated. The study team will certify participant-facing coordinators by having them role play the surrogate approach, consent, and FST orientation. For each enrollment for a period of time at site initiation, coordinators will call study staff at the coordinating center to talk through their plan for intervention deployment, so that the study staff can provide feedback as needed. Coordinators will then meet biweekly throughout the study to reinforce the protocol and address issues.

The study team will monitor the surrogates’ real-time progress using customized reporting features in the FST administrative dashboard. If surrogates do not complete expected portions of the FST, the study team will offer encouragement and technical support to ensure completion before each of the goal timepoints. Similarly, study team will facilitate scheduling of family meetings per protocol, to help ensure these happen at the goal timepoints. We will quantify the amount of staff time needed for orientation of surrogates to the FST in order to gain insight into future uptake.

### 2.8. Retention strategies

We will use several strategies to maximize retention for ascertainment of trial outcomes at 3 and 6 months. These methods included short measurement instruments, telephone follow-up, and emailed and mailed surveys, with a dedicated long term follow-up team rather than in-person visits, and incremental compensation after follow-ups.

### 2.9. Randomization

Following completion of the primary surrogate’s baseline survey, study staff will randomize the enrolled patient into either the intervention group or the control group using a 1:1 randomization scheme in REDCap provided by the coordinating site. Computerized procedures will randomize participants at the patient-level, stratified by site, with 50% probability to each trial arm. Randomization procedures are designed to ensure allocation concealment prior to assignment. With 7 enrolling sites, we anticipate each site will enroll 53 patients, with 26–27 assigned to the standard care arm and 26–27 to the intervention arm. Every two additional enrollments will be randomized in the order of which arm is first assigned. To create the randomization scheme, our study statistician will use Stata to create 53 observations and then generate a random number from uniform distribution. The first 26–27 observations will be assigned to standard care and the latter 26–27 to intervention. The records will be then sorted by random number.

### 2.10. Standard care arm

Participation in the trial will not affect access to palliative care, ethics, or other resources available at all study sites. Participants randomized to the usual care arm will receive communication and support at the discretion of the treating clinicians.

### 2.11. Data collection

We will establish baseline levels of surrogate psychological distress and unmet care needs, using the HADS and NEST scales [24,25]. We will also collect detailed demographic and disease data, frequency of multidisciplinary family meetings, bedside updates, palliative care consults, and encounters between the family and the social worker and pastoral care providers, gleaned from chart review. We will use web analytics to quantify how often surrogates accessed the FST and which sections of the tool they viewed. Primary and secondary outcome measures will be administered by phone or email to surrogates at 3 and 6 months. All outcome assessments will be blinded to ensure unbiased evaluation [25,26].

### 2.12. Outcomes

We will measure the intervention’s effect on four domains of outcomes: patient clinical outcomes, surrogates’ long-term psychological outcomes, decision/communication quality, and end-of-life healthcare utilization. The primary outcome is patient centeredness of care, measured at 3-month follow-up using the 12-item Patient-Perceived Patient-Centeredness of Care Scale (PPPC) modified for surrogates [26–28]. Secondary outcomes are summarized in Table 2.

### 2.13. Statistical analysis plan

To assess the effectiveness of randomization, we will compare distributions of baseline characteristics for patients and surrogates between the two groups (FST intervention and standard care). For the superiority hypotheses (e.g., efficacy outcomes for the FST), all analyses will be performed under the intention to treat principle. We will follow best practice recommendations to perform non-inferiority analyses under the per-protocol principle. To compare the primary outcome between the two trial arms, we will employ linear mixed effect modeling. For each of the secondary outcome measures, depending on the distribution, a linear mixed effects model or a generalized linear mixed effects model with appropriate link function will be used to compare the difference between intervention groups. Likelihood ratio tests will be used for testing the intervention effect. To confirm our assumption of minimal clustering effects, we will calculate intraclass correlation coefficients for each outcome.

We conservatively estimate a 20% loss to follow up rate, meaning the total effective sample size will be 296 patients. All aforementioned power and sample size estimations are based on this effective sample size, with 1.5 surrogates per patient. For the analysis based on the per-protocol principle, an inverse-probability weighting method from the attrition model will be used. Baseline covariates and 3-month non-missing covariates will be included in the 3-month attrition model, while baseline, 3-month, and 6-month non-missing covariates will be included in the 6-month attrition model to maximize the accuracy of the estimation.

### 2.14. Ethics and dissemination

The institutional review board of the University of Pittsburgh and the associated hospitals approved the project. The leadership of each participating ICU approved the project. The study is overseen by an institutional Data Safety Monitoring Board (DSMB), who will meet regularly and monitor participant safety and study progress. Surrogates of eligible patients will be consented for enrollment. We will present findings at national meetings and submit findings to peer-reviewed journals.

## 3. Discussion

We wish to highlight several key methodological decisions about the trial design considerations including 1) determining the optimal type of randomized trial, 2) selection of outcome measures, and 3) ensuring and monitoring of intervention fidelity.

We opted to conduct carefully controlled efficacy trial rather than a pragmatic trial. Our rationale was: Existing guidelines for intervention development recommend that carefully controlled efficacy trials precede pragmatic trials, in order to first establish that the intervention is efficacious when delivered with high fidelity [29,30]. In addition, engagement with the leadership of our study sites revealed that they want evidence of efficacy before undertaking the staff training and system-level changes that would be required for a “real world” pragmatic trial involving complete implementation of the intervention by clinical staff.

We opted to randomize at the patient-level versus conducting an ICU-level cluster RCT We did this for several reasons. First, while there is a risk of contamination with this design because consented clinicians may have several intervention patient-family dyads enrolled under their care, it is likely very low because the intervention primarily targets individual surrogates. Interviews with physicians during the pilot RCT confirmed that the intervention did not alter the care they delivered to the control arm, and due to staffing patterns in study ICUs, individual physicians will only care for 3–4 intervention patients during the study period, which makes it very unlikely that passive learning from chronic exposure to the intervention would change control group care practices. Finally, an adequately powered cluster RCT would be infeasible, in terms of financial and institutional resources.

Because the intervention is comprised of multiple components that work together to address multiple problems across multiple domains, we selected a wide range of outcomes. Specifically, we are interested in the intervention’s impact on patients’ clinical outcomes, surrogates’ psychological outcomes, decision and communication quality and end-of-life healthcare utilization, so we selected validated and responsive outcome measures to test hypotheses for each of these domains. The primary goal of this intervention is to increase the patient-centeredness of care, and the degree to which care aligns with patient goals. Thus, the primary outcome is a robust metric of patient centeredness of care, the PPPC, modified for surrogates [26,31–36]. We selected the PPPC because it has solid psychometric properties including responsiveness to change, and it is reported to be one of the best instruments to measure whether ICU care is tailored to patient individual values and preferences [26–28,37–41]. We selected 6-month survival as a safety outcome because we wish to address any concerns that robustly supporting surrogates facing EOL decisions might lessen survival over 6 months. Despite recent evidence that palliative approaches to care may prolong survival, some may fear the opposite is true [42,43]. We adequately powered the study to “rule out” increased mortality in the intervention arm through non-inferiority testing. We acknowledge that there is legitimate debate about whether a finding of shorter survival with better quality of dying in one arm would be a preferable outcome. Therefore, we believe our responsibility is to design our study to provide the data necessary to inform DSMB oversight and a broader public discussion.

To establish and maintain intervention fidelity in this efficacy trial, we developed a rigorous and multifaceted intervention fidelity monitoring plan according to recommendations from the NIH Behavior Change Consortium. In addition, in a novel approach to intervention fidelity monitoring, we took advantage of an interactive, virtual tool being part of this intervention, and built intervention fidelity monitoring components into its design. In this way, study staff will receive real-time accurate feedback about surrogate progress on the FST, allowing for timely interventions from them to increase fidelity. Study staff themselves participated in planning and coordinating interdisciplinary family meetings at the set meeting times, in addition to nudging clinical teams and surrogates to complete their parts in the intervention. We also used more standard methods of IFM, following approaches that we have used in the past, which have allowed us to achieve greater than 95% fidelity to a complex behavioral intervention in past clinical trials [44,45]. These processes included: 1) multimodal orientation and training of interventionists, 2) detailed fidelity monitoring using a combination of clinician-reported checklists and chart review, and 3) ongoing feedback and support of clinical teams.

One limitation is that we do not have attention control. Therefore, we cannot exclude the possibility that any positive effect might be due not to the specific active ingredients of the digital intervention, but to the higher degree of interaction with research staff to help onboard them to the study. We think this is exceedingly unlikely given that the research staff will not be trained to deliver emotional support and will be instead trained to be research coordinators.

## 4. Conclusion

Our primary goal when designing this intervention to improve patient centeredness of care, surrogate long-term psychological outcomes, communication and decision quality, and end-of-life healthcare utilization, was to create something that was feasible and scalable across settings. For this reason, we combined interactive digital support of families with systems level care redesign to improve existing communication processes in a manner that does not add new members to a care team or utilize significant new resources. We look forward to testing the impact of this novel approach across several key domains.

## Figures and Tables

**Table 1:** Content of the family support tool at the three prompted time points, each of which use videos and animation, as well as narration by family members of prior intensive care patients. Each section takes about 25 min to complete. FST = Family Support Tool. ICU = Intensive Care Unit.

FST section	Section 1: about the ICU	Section 2: getting ready to talk to the healthcare team	Section 3: your loved one’s values and treatment options	Section 4: getting ready for your next meeting with the healthcare team
Timing of completion	On enrollment	Before first family meeting (days 0–2)	Before first family meeting (days 0–2)	Before second family meeting (days 5–9)
Associated interventions	Orientation by study staff	Repeated at each section/step, and then weekly: - Proactively scheduled interdisciplinary family meeting - One-page summary sheet given to surrogates, physicians, and nurses, after completion of each FST section and prior to each family meeting		
Framework informing FST component Cognitive Emotional Decision-Making (CEDM) Framework*	1. Video messages of support from families 2. Self-care tips 3. Brief videos of stories from other families about their coping strategies	1. Tips from families about being a surrogate and talking to clinicians in ICU	1. Stories of other families’ experiences 2. Video of encouragement from clinicians and families 3. Tips from families about surrogacy	1. Describing three pathways of care (full life support, time-limited trial, comfort focused care) 2. Eliciting surrogate’s prognostic expectations and “treatment leanings”
Ottawa Decision Support Framework^€^	1. Videos and animations explaining ICU machines, personnel, and routines 2. Links to other online resources	1. Video of family meetings 2. Interactive question prompt list 3. Interactive exercises about patient values and preferences 4. Eliciting surrogate’s prognostic expectations	1. Describing three pathways of care (full life support, time-limited trial, comfort-focused care) 2. Interactive question prompt list	

* Cognitive Emotional Decision-Making (CEDM) Framework: focus on influence of emotion on decisions, coping, cognitive and emotional factors, and decisions as coping process [14].

€ Ottawa Decision Support Framework: tools to assess decisional needs, provide decision support interventions, and evaluate decision outcomes [18].

**Table 2:** Outcome domains and the methods through which each will be assessed. ICU = Intensive Care Unit.

Domain	Instrument	Data source	Timepoint for collection
Patient Clinical Outcomes, Measures of Patient Centeredness of Care	Patient-Perceived Patient-Centeredness of Care Scale (PPPC)*	Surrogate (s)	3 months
	Needs of Social Nature, Existential Concerns, Symptoms, and Therapeutic Interaction (NEST)‡	Patient, surrogate (s)	Hospitalization Day 5
	Goal concordance of care^¶^	Surrogate (s)	3 months
	Quality of Death and Dying (QODD)^†^	Surrogate (s)	6 months
	Survival to discharge	Chart abstraction	6 months
	Duration of survival from enrollment to 6-months	Chart abstraction	
	Days alive at outside facility	Chart abstraction	
	Katz Index of Independence in Activities of Daily Living (patient’s functional status)^††^	Chart abstraction	
	Proportion of patients with comfort-focused care	Chart abstraction	
	Proportion of patients with new DNR during hospitalization	Chart abstraction	
	Proportion of patients enrolled in hospice during hospitalization	Chart abstraction	
	Time to hospice	Chart abstraction	
Surrogate Psychological Outcomes and ICU Experiences	Hospital Anxiety and Depression Scale (HADS)^‡‡^	Surrogate (s)	During admission
	Clinician-family conflict^₸^	Surrogate (s)	Hospitalization Day 2, Day 7
	Perceived effectiveness of FST^₸₸^	Surrogate (s)	Hospitalization Day 2, Day 7
	Family Satisfaction in the ICU (FS-ICU)^¶¶^	Surrogate (s)	3-months
	Impact of Events Scale-revised (IES-R, risk of post-traumatic stress disorder)^β^	Surrogate (s)	6-months
Patient Healthcare Utilization	ICU length of stay, hospital length of stay, duration of mechanical ventilation, index hospital costs	Chart abstraction	Throughout hospitalization
	Surrogate interviews to identify post-discharge encounters and costs	Surrogate (s)	Day 90, Day 180
Communication and Decision Quality	Clinician-Surrogate Concordance Scale (CSCS)***	Surrogate (s)	Hospitalization Day 2, Day 7
	Preparation for Decision-Making Scale^†††^	Surrogate (s)	Hospitalization Day 2, Day 7
	Decisional Conflict Scale (DCS), Decision Self-efficacy Scale^‡‡‡^	Surrogate (s)	Hospitalization Day 2, Day 7
	Quality of Communication (QOC) Scale**	Surrogate (s)	3-months

* PPPC is a 12-item instrument that measures the patient-centeredness of care. It has been found to be one of the two best instruments to measure this construct, and has demonstrated validity and reliability when used by surrogates [26–28,39,40].

† QODD is a 19-item instrument with established psychometrics and successful use with ICU surrogates [46].

‡ NEST, or the adapted Needs of Social Nature, Existential Concerns, Symptoms, and Therapeutic Interaction [24,25] scale, is designed for ICU use; it is a 13-item instrument developed to identify unmet social, emotional, physical, and care-system needs in serious illness.

¶ Goal-concordance of care will be tested using an 8-item composite measure of goal-concordant care, in addition to the After-Death Bereaved Family Interview [3,47–49].

†† Patient functional status will be assessed using the Katz Index of Independence in Activities of Daily Living, a validated and widely-used scale [50].

‡‡ HADS is a 14-item assessment with subscales for anxiety and depression, with established reliability and validity among ICU surrogates that is recommended by consensus guidelines for use among ICU surrogates [32–35]. Each domain has a score range of 0–21 with the following interpretation: 0–7 normal, 8–10 borderline abnormal and 11–21, abnormal [32].

¶¶ Satisfaction with ICU care will be assessed using the Family Satisfaction in the ICU (FS-ICU) instrument at 3-month telephone follow-up of surrogates. The FS-ICU is a 24-item scale concerning satisfaction with care, communication, and decision-making in the ICU [15].

₸ A brief survey (measured by both surrogates and physician) will be used to determine the level of family-clinician conflict during index hospitalization.

₸₸ A brief survey asking (intervention only) participants about the perceived effectiveness of the FST intervention, during hospitalization.

β IES-R [33,51,52] is a valid, reliable, and responsive 22 -item instrument measuring symptoms of avoidance and intrusive thoughts. It is a 15-item tool measuring total stress (score range of 0–75) with subscales for intrusiveness (score range 0–35) and avoidance (score range 0–40). Total stress score is interpreted as follows: 0–8 subclinical range, 9–25 mild range, 26–43 moderate range, and 44+ severe range. A score of ≥30 indicates a high risk of post-traumatic stress disorder. The IES is a valid, reliable and responsive instrument used among ICU surrogates measuring risks of post-traumatic stress disorder (PTSD) [33,51,52].

** QOC is a 13-item scale measuring clinician QOC with good internal consistency, strong evidence of reliability and validity and established responsiveness to change [53,54].

*** CSCS is a validated, reliable, and responsive system for scoring the concordance of clinician and surrogate understanding of prognosis. It was developed by our team [55–57]. The single item CSCS has excellent test-retest reliability (*r* = 0.91). It has established criterion validity and responsiveness to change.

††† Preparation for Decision-Making Scale is a 10-item validated, consistent and reliable tool for testing surrogate preparedness to make decisions [58].

‡‡‡ Surrogates’ clarity about preferences will be assessed using the “informed” and “values clarity” subscales, 6 items out of the 16-item Decisional Conflict Scale (DCS) [55,59–61]. The scale has established responsiveness to change, test-retest reliability (*r* = 0.81), internal consistency (α = 0.92), and discriminant validity. DCS is a reliable, validated and responsive 16-item tool assessing surrogate clarity about patient values and preferences [55,59–61].

## Data Availability

No data was used for the research described in the article.
